# The role of digital health in pandemic preparedness and response: securing global health?

**DOI:** 10.1080/16549716.2024.2419694

**Published:** 2024-10-22

**Authors:** Chisom Ogochukwu Ezenwaji, Esther Ugo Alum, Okechukwu Paul-Chima Ugwu

**Affiliations:** a Department of Sociology and Anthropology, University of Nigeria Nsukka, Enugu, Nigeria; b Department of Research and Publications, Kampala International University, Kampala, Uganda

**Keywords:** Digital health technologies, COVID-19, telehealth, pandemic preparedness, health surveillance

## Abstract

**Background:**

Digital health technologies, such as mobile applications, wearable devices, and electronic health record systems, have significantly enhanced global health security by enabling timely data collection and analysis, identifying infectious disease trends, and reducing infection risk through remote services.

**Objective:**

This study assesses the role of digital health in pandemic preparedness and global health security response. It examines the application of digital health to early detection, surveillance, and data management in patient care.

**Methods:**

We gathered data from scholarly articles published between 2019 and 2024 (found in PubMed, Science Direct, Google Scholar, and Web of Science), reports from the WHO, and case studies of recent pandemics. Topics discussed include digital health technologies, their use, benefits, and issues. We paid special attention to gathering the informed opinions and perspectives of specialists from various fields, including public health, technology, and government. The commentary synthesises these findings to offer suggestions for incorporating digital health into future pandemic preparedness and response.

**Results:**

Digital tools improve communication, combat fake news, and reach the public, but data protection and public health remain challenges. Integration requires extensive research and collaboration between governments and the private sector.

**Conclusion:**

The COVID-19 outbreak demonstrated the importance of digital technology in outbreak management, patient care, communication, and data sharing. As the world transitions into the post-pandemic phase, it will be important to build on these innovations and prepare for the challenges ahead in order to strengthen healthcare systems for future pandemics.

## Paper Context

**Main finding:**

Digital health technology services like mobile health, big data analytics, and artificial intelligence are essential components of pandemic preparedness and response.

**Added knowledge:**

This commentary integrates COVID-19 lessons and examines digital health’s development in the global health security system, highlighting shortcomings and opportunities for further discoveries.

**Global health impact for policy and action:**

This commentary highlights priorities for digital health in public health preparedness and makes the case for investment in infrastructure, workforce, and technology access. It highlights the need for international collaboration in combating global health threats and advancing a collaborative, equitable strategy for pandemic readiness.

The COVID-19 pandemic has shed an exponential spotlight on the critical need for strong, well-funded plans to prepare for and respond to pandemics [1]. Digital health technologies are currently at the forefront of the discussion about consequences and potential threats because they represent revolutionary approaches to changing global health security [2]. The early detection and surveillance of diseases no longer rely on antiquated methods; digital health technologies have made it possible to do all such tasks with a compassionate approach. Mobile health apps, wearable devices, and electronic health records (EHRs) allow for the collection of real-time data analysis, which can assist in recognising new trends in infectious diseases. Early warning systems using big data analytics detect uncommon patterns associated with outbreaks and allow rapid public health responses [3,4]. Throughout the COVID-19 epidemic, digital tools like contact-tracing apps and syndromic surveillance systems have been critical in tracking (and preventing) its spread [5]. Telehealth has been a lifesaver during lockdowns and social distancing. It has enabled remote consultations, taken the burden off overwhelmed healthcare facilities, and reduced the risk of infection transmission. Telehealth has also expanded access to healthcare in underserved areas, bridging the healthcare equity gap. The rapid adoption of telehealth during the pandemic has shown us that it has the potential to ensure healthcare resilience in times of crisis and beyond [6].

Digital health technology has been crucial in combating the COVID-19 outbreak in Africa. South Africa used technologies such as SMS-based solutions, mobile health applications, telemedicine, and artificial intelligence for illness screening, surveillance, and virtual healthcare services [7]. Uganda experienced a rise in telehealth modalities, such as tele-consultation and tele-medicine, to ensure the continuity of health care under movement limitations [8]. These digital technologies possess the capacity to enhance pandemic preparedness, response, and recovery throughout African nations [9]. They facilitate swift identification, diagnosis, and management of COVID-19 cases, in addition to promoting equitable vaccination distribution [9]. Nonetheless, obstacles persist, encompassing infrastructural, technological, and regulatory impediments [7]. Notwithstanding these challenges, digital health technologies present intriguing possibilities for enhancing healthcare delivery and pandemic management in Africa.

Digital health technologies have been crucial in addressing the COVID-19 pandemic in advanced countries. Kalhori and colleagues examined the impact of digital health technologies used in the COVID-19 pandemic control in ten selected countries with the highest prevalence (Italy, Spain, the United States, France, the United Kingdom, Iran, China, the Netherlands, Germany, and Belgium). According to them, telemedicine was the most predominant technology, followed by electronic health records, artificial intelligence, big data, and the Internet of Things [10]. In a scoping review of literature, Tilahun et al. [11] reported that these technologies served multiple objectives, including patient screening and management, exposure reduction, disease simulation, and healthcare provider assistance. Digital learning modules, geographic information systems, and mobile applications for self-care and patient monitoring were also significant in COVID-19 pandemic control [10]. Vargo et al. [12] identified 28 distinct technological devices used by eight diverse populations, predominantly medical professionals, for 32 different activities. The implementation of these technologies yielded 35 distinct results, encompassing enhanced patient outcomes and reduced outbreak impact [12].

Communication is key during a pandemic. Digital platforms have enabled public health authorities to disseminate information to the public in real time, counter misinformation, and get people to comply with health guidelines. Public health authorities have used social media, websites, and mobile apps to educate the public on preventive measures, vaccine information, and updates on the pandemic situation. These platforms have also enabled community engagement to provide feedback and address public concerns [13].

Digital health technologies have brought together multiple data sources for pandemic response. Interoperable health information systems facilitate the sharing of data among various sectors such as healthcare, public health, and research. This is key to coordinated responses, resource allocation, and informed decision-making [14]. During the COVID-19 pandemic, integrated data systems helped track hospital capacity, vaccine distribution, and epidemiological trends.

Despite the potential of digital health, there are challenges and ethical considerations to address. As we use more digital tools, data privacy and security become top priorities. There’s concern about the confidentiality and protection of personal health information [15]. Ensuring equitable access to digital health technologies is another big issue, as digital literacy and infrastructure disparities can worsen existing health inequities. And we should not rely too much on digital tools, forgetting to strengthen traditional public health infrastructure and workforce capabilities.

Digital health should be part of global health security strategies. To make the most of these technologies, allocate resources to research, capacity building, and infrastructure. The government, private sector, and international organisations should collaborate to innovate and share digital health resources. Digital health technologies face infrastructural, financial, and regulatory constraints. Experts recommend formulating guidance rules, standardising deployment procedures, and cultivating public–private partnerships to enhance effectiveness during pandemics [7]. Developing guidelines for data governance and interoperability, along with other recently highlighted areas like ethics, is crucial as we continue to invest in digital health technologies to ensure their sustainable role in patient care. In their research on utilizing digital health data governance to safeguard vulnerable populations in low- and middle-income countries, Tiffin and colleagues proposed four essential domains within this framework: ethical oversight and informed consent procedures, data protection via access controls, sustainability of ethical data usage, and adherence to pertinent legislation [16]. To build public trust and realize the full potential of digital health, these frameworks must consider patient privacy rights, data security, and inclusive accessibility for all.

During the COVID-19 pandemic, digital health was an essential tool for preparedness and response in areas of surveillance, patient management, communication, and outreach through data integration. With the global community transitioning into a post-COVID-19 era, it is crucial to catalyse these gains and tackle challenges arising from them in order to sustain health systems that are able to withstand recurrent pandemics. The value of the information a digital health technology delivers in relation to its implementation setting determines its efficacy. Adherence to evaluation through evidence-based medicine and complex systems paradigms will be essential for the safe and effective application of digital health technologies in response to COVID-19 and future pandemics. Digital health is an integral strategy for ensuring global health security, as it makes any future pandemic less likely to occur and much more manageable if one does.
